# Behind the mask of relapsing bimodal encephalitis: herpesvirus 7 and Epstein-Barr virus associated with Hashimoto’s encephalopathy: a case report

**DOI:** 10.3389/fimmu.2026.1782631

**Published:** 2026-05-29

**Authors:** Yinxue Xing, Jing Wang, Xiaoqing Li, Xiaoqian Yin

**Affiliations:** 1 Neurology Department, Xingtai People’s Hospital, Xingtai, Hebei, China; 2 Imaging Department, Xingtai People’s Hospital, Xingtai, Hebei, China

**Keywords:** bimodal encephalitis, Epstein-Barr virus, Hashimoto’s encephalopathy, human herpesvirus 7, psychiatric disorder

## Abstract

**Background and purpose:**

Relapsing bimodal encephalitis in adults remains poorly characterized. We describe a case of relapsing viral encephalitis followed by secondary autoimmune-mediated encephalitis and explore its potential underlying mechanisms.

**Case description:**

A previously healthy adult female initially presented with fever and headache; brain magnetic resonance imaging (MRI) showed punctate white matter hyperintensities, and electroencephalogram revealed background slowing with intermittent δ waves. Initial cerebrospinal fluid (CSF) analyses were suggestive of viral encephalitis. Although no pathogen was identified by CSF metagenomic next-generation sequencing (mNGS), she responded favorably to empirical antiviral therapy. 22 days after discharge, she was readmitted with decreased responsiveness, hypersomnia, and acute psychosis. Concurrent MRI revealed progressive white matter lesions. CSF analysis demonstrated oligoclonal bands restricted to the CSF, while autoimmune encephalitis antibody panels were negative. Serum autoantibodies (anti-SSA/SSB, anti-thyroglobulin, and thyroid peroxidase) were elevated. Considering probable autoimmune encephalitis, intravenous immunoglobulin and methylprednisolone were administered, leading to clinical and serological remission with radiological improvement. After 22 months, the patient relapsed with similar clinical manifestations, beginning with fever and headache, followed by decreased responsiveness, and subsequently developed an acute mental disorder. Repeat CSF mNGS detected human herpesvirus 7 (HHV-7) and Epstein-Barr virus (EBV), accompanied by new white matter lesions and recurrent thyroid autoantibodies. The patient responded favorably to the same treatment. At one-month follow-up, the patient developed hyperthyroidism.

**Conclusion:**

HHV-7 and EBV with long latency are likely associated with a cascade of autoimmune encephalitis, presenting as relapsing bimodal encephalitis. Thyroid autoantibodies—rather than conventional neuronal antibodies—appear central to the autoimmune phase, consistent with Hashimoto’s encephalopathy.

## Introduction

Existing evidence indicates that patients with relapsing post-herpes simplex encephalitis often test negative for herpes simplex virus (HSV) via polymerase chain reaction (PCR) in cerebrospinal fluid (CSF) yet demonstrate the presence of N-methyl-D-aspartate receptor (NMDAR) antibodies and other unidentified autoantibodies (1, 2). These autoantibodies are implicated in the pathogenesis of autoimmune encephalitis, leading to cognitive impairment, seizures, and abnormal behaviors in affected individuals. Notably, a substantial proportion of relapses in appropriately treated HSV encephalitis cases are now recognized to result from autoimmune-mediated mechanisms rather than viral persistence (3). This manifestation of two distinct peaks in the course of encephalitis is always referred to as bimodal encephalitis, which typically presents with an initial peak of viral encephalitis followed by a secondary peak of autoimmune encephalitis (4). Japanese encephalitis can also cause autoimmune encephalitis during the recovery period, manifesting as a clinical course of bimodal encephalitis (5). Beyond HSV and Japanese encephalitis virus, few other viral triggers of bimodal encephalitis have been documented in the literature.

HSV has been implicated not only in secondary immune-mediated encephalitis but also in the development of secondary Hashimoto’s thyroiditis, which is marked by autoantibodies to thyroglobulin (Tg) and thyroperoxidase (TPO) (6). The clinical finding of anti-Tg/TPO antibodies suggesting a possible existence of Hashimoto’s encephalopathy (HE) or a wider neurological spectrum (7). Although elevated thyroid autoantibodies (anti-TPO or anti-Tg) are required for the diagnosis of HE and are found in most patients—with reported frequencies of 100% for anti-TPO and 48% for anti-Tg—they lack disease specificity, as raised anti-TPO antibodies are present in about 10% of the general population (8). Moreover, while antithyroid antibodies and circulating immune complexes have been identified in the CSF of HE patients (9), the sensitivity and specificity of these antibodies as diagnostic markers for HE remain unclear. The presence of these autoantibodies merely indicates an underlying autoimmune status rather than directly pointing to the etiology of HE. Although Hashimoto’s thyroiditis has been reported following HSV infection, there is no established pathological basis for these antibodies to serve as a bridge leading to secondary Hashimoto’s encephalopathy. To date, no cases of secondary Hashimoto’s encephalopathy associated with HSV or other herpesviruses have been reported.

## Case description

### First hospitalization

A 22-year-old woman presented with a 4-day history of high-grade fever (maximum temperature 39.3 °C), headache, nausea, and vomiting. Initial evaluation revealed marked leukocytosis (24.94×10^9/L) with neutrophilic predominance (92.7%), while chest radiography showed no abnormalities. Despite 2 days of intravenous penicillin (6 million units twice daily), her symptoms persisted, prompting referral to our hospital. Past Medical History: Miscarriages at 2 months and 8 months prior; no psychosocial history or other medical conditions. No history of smoking, alcohol use, toxin exposure, or other substance abuse. No family history of diseases. Occupation: Homemaker (unemployed).

On admission, the patient exhibited marked neck resistance without other neurological abnormalities. Blood tests showed normal coagulation function, complete blood count (CBC), glucose, liver function, homocysteine, renal function, procalcitonin, and thyroid function, except for elevated C-reactive protein (CRP) (33.12 mg/L, normal < 6 mg/L) and slightly reduced potassium (3.13 mmol/L). Routine urine and stool tests were also normal. Serological screening for hepatitis B virus (HBV), hepatitis C virus (HCV), syphilis, and human immunodeficiency virus (HIV) was negative.

The initial lumbar puncture revealed: (1) elevated CSF pressure (215 mm H_2_O); (2) increased white blood cell (WBC) count (93/μL, normal <8/μL), with 20/μL polymorphonuclear and 73/μL mononuclear cells; (3) high CSF protein (0.684 g/L, normal 0.15-0.45g/L), with normal glucose, chloride, adenosine deaminase (ADA), and lactate dehydrogenase (LDH) levels; (4) CSF cytology under the microscope showed numerous WBCs (>100), predominantly lymphocytes (59%), neutrophils (25%), and monocytes (16%, including 2% activated) (**Figure 1A**); (5) no bacteria, viruses, mycobacteria, fungi, or parasites were detected in CSF culture or metagenomic next-generation sequencing (mNGS). Brain magnetic resonance imaging (MRI) demonstrated punctate T2-FLAIR hyperintensities in left periventricular and hemisphere white matter (**Figures 2A, B**). Electroencephalogram (EEG) revealed background slowing with insertable δ slow waves localized to the left frontal pole and anterior mid-temporal region (**Figure 3A**).

**Figure 1:**
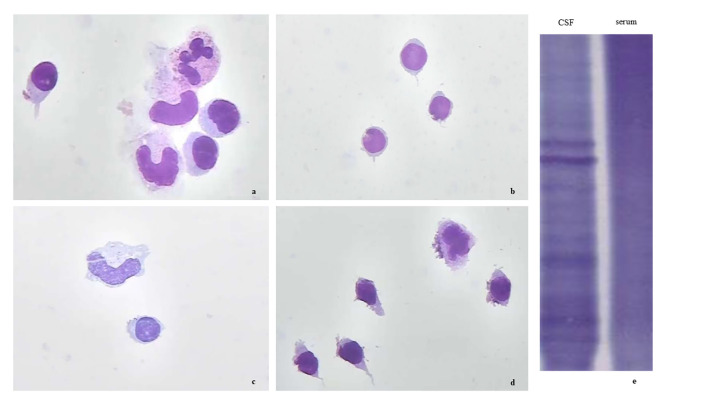
**(A–D)** displayed the CSF cytology results under the microscope during four lumbar punctures. **(E)** revealed the oligoclonal bands only existed in CSF in the second hospitalization.

**Figure 2:**
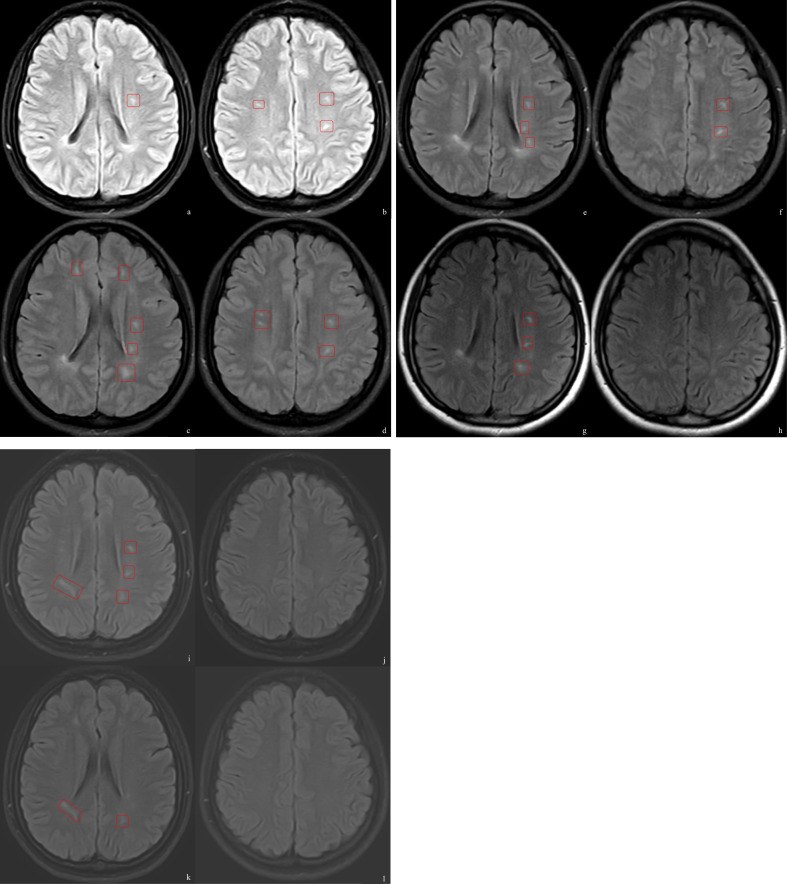
**(A–L)** showed the dynamic change of brain MRI T2 FLAIR images of the patient during three hospitalizations and the follow-up after discharge. The punctate hyperintensities in the periventricular and hemispheric white matter are marked in red boxes.

**Figure 3:**
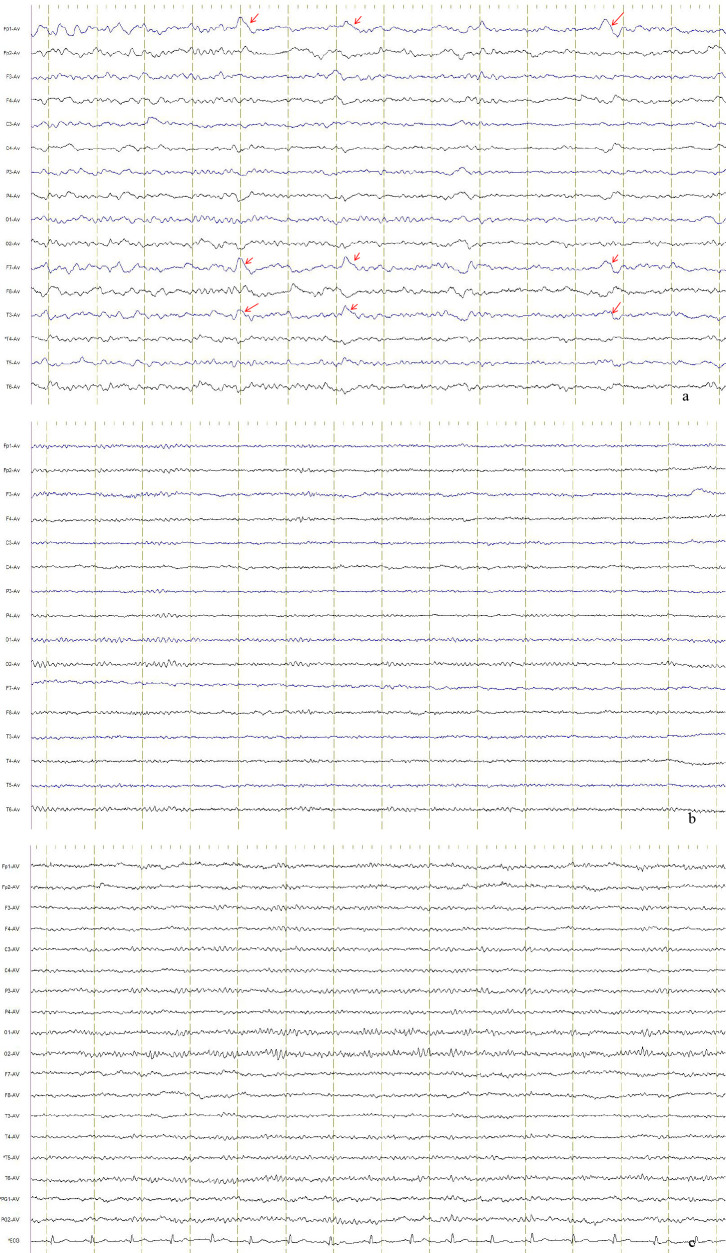
**(A–C)** showed the background of electroencephalograms during three hospitalizations. **(A)** showed the background rhythm of the electroencephalogram in the first hospitalization slows down to 8 Hz with insertable δ slow waves localized to the left frontal pole and anterior midtemporal region (the δ slow waves were marked by red arrows). **Figure 2B, C** showed the normal electroencephalograms in the second and third hospitalizations.

The patient received intravenous ganciclovir, cefotaxime, mannitol, and glycerol fructose. High−grade fever resolved by day 3 and normalized by day 7, at which point cefotaxime was discontinued. Headache had sufficiently improved by day 7 to allow cessation of mannitol and glycerol fructose. The second lumbar puncture by day 11 revealed normalized pressure (120 mm H_2_O) and improved CSF parameters: WBC count dropped to 30/μL (1 polymorphonuclear, 29 mononuclear), normal biochemistry, and reduced cytology (77 lymphocytes, 5 monocytes) (**Figure 1B**). On day 14, the patient recovered and was discharged with continued oral ganciclovir for consolidation antiviral therapy.

### Second hospitalization (22 days after discharge)

After 22 days, the patient was readmitted with new-onset psychiatric symptoms, beginning with 9 days of decreased responsiveness and 2 days of hypersomnia. In the 21 hours preceding admission, she developed acute mania characterized by psychomotor agitation, perseverative speech (repetitively uttering the same phrases), and shouting the names of absent family members, accompanied by irritability, hallucinations, and impaired impulse control.

On admission, her temperature was normal, and there were no signs of infection. CBC revealed a leukocyte count of 17.6×10^9/L, neutrophil count of 14×10^9/L, monocyte count of 1.9×10^9/L, neutrophil percentage of 79.8%, and platelet count of 374×10^9/L; while other blood tests, including glucose, electrolytes, ketones, liver and renal function, thyroid function, coagulation, erythrocyte sedimentation rate (ESR), and CRP, were normal. A brain CT scan showed no abnormalities.

The third lumbar puncture showed CSF pressure of 200 mm H_2_O, WBC count of 17/μL, normal biochemistry, and 58 lymphocytes with 6 monocytes on cytology (**Figure 1C**). Brain MRI revealed increased punctate hyperintensities in periventricular and hemisphere white matter (**Figures 2C, D**). The second EEG displayed a normal background (**Figure 3B**). A cardiac ultrasound foam test was negative.

The patient was initially sedated with intramuscular phenobarbital. Given clinical findings suggestive of probable autoimmune-mediated encephalitis, intravenous immunoglobulin (IVIG) therapy (0.4 g/kg/day) was administered for 5 days after informed consent. Parallel immunological evaluations in CSF and serum had been carried out. By day 4, the acute mania improved significantly, allowing phenobarbital cessation, though psychomotor slowing and memory impairment persisted. By hospital Day 6, the immunological results were obtained as follows: (1) markedly elevated intrathecal immunoglobulin synthesis including IgG: 64.36%, IgA: 64.35%, and IgM: 87.18% (normal <10%); (2) elevated CSF IgG index: 1.74 (normal ≤0.7); (3) increased 24-hour CSF IgG synthesis rate: 12.99 mg (normal <7 mg); (4) oligoclonal bands only existed in CSF (**Figure 1E**); (5) significant elevation of anti-SSA (56.4 IU/mL), anti-SSB (123 IU/mL), and antinuclear antibodies (123 IU/mL, normal <24) in serum; (6) significant elevation of anti-thyroglobulin (anti-Tg) (1407 IU/mL, normal <90) and anti-thyroid peroxidase (anti-TPO) (657 IU/mL, normal <30) antibodies in serum; (7) negative findings for other systemic autoantibodies including anti-double-stranded DNA, anti-cardiolipin, anti-SM, anti-neutrophil cytoplasmic antibodies (ANCA), anti-myeloperoxidase, anti-glomerular basement membrane, anti-JO, anti-SCL-70, and thyroid stimulating receptor antibodies in serum; (8) negative for all tested neural autoantibodies in CSF and serum including anti-N-methyl-D-aspartate (NMDA) receptor, anti-α-amino-3-hydroxy-5-methyl-4-isoxazolepropionic acid 1 (AMPA1) receptor, anti-AMPA2 receptor, anti-leucine-rich glioma inactivated protein 1 (LGI1), anti-contactin-associated protein-like 2 (CASPR2), anti-γ-aminobutyric acid B (GABA B) receptor, anti-dipeptidyl-peptidase-like protein-6 (DPPX), anti-metabotropic glutamate receptor 5 (mGluR5), anti-glutamate decarboxylase 65 (GAD 65), anti-myelin oligodendrocyte glycoprotein (MOG), and IgLON5 antibodies. Based on the findings of positive systemic autoantibodies and intrathecal immune activation, intravenous methylprednisolone was initiated at 500 mg/day for 3 days, then tapered stepwise (240 mg, 160 mg, 80 mg) every 3 days. On day 15, piracetam was added to alleviate psychomotor slowing and memory impairment; however, the patient developed transient manic episodes, which resolved upon discontinuation of the medication. A third EEG was also normal. A third brain MRI revealed reduced punctate hyperintensities in the white matter (**Figures 2E, F**). The therapy was transitioned to oral methylprednisolone (40 mg/day) by day 18, and the patient was discharged on day 21 with a tapering schedule of 4 mg every two weeks.

At the three-month follow-up visit, the patient had fully recovered from the previous psychomotor slowing and memory impairment. Previously elevated antibodies, including anti-SSA/SSB, anti-thyroglobulin, and anti-thyroid peroxidase antibodies, seroconverted to negative. The punctate hyperintensities of hemisphere white matter on MRI were also gradually dissipated (**Figures 2G, H**). Given the seroconversion of anti-SSA/SSB antibodies and the absence of sicca symptoms (xerophthalmia or xerostomia), a previously planned salivary gland biopsy for suspected Sjögren’s syndrome was canceled at the patient’s request.

### Third hospitalization (22 months after discharge)

After a 22-month period of remission, the patient was readmitted with a 7-day history of low-grade fever (peak 37.5 °C) and headache. She experienced intermittent decreased responsiveness 4 hours before admission.

The fourth lumbar puncture revealed: (1) CSF pressure of 165 mm H_2_O; (2) WBC count of 36/μL (1 multinucleated, 35 mononucleated); (3) normal CSF biochemistry; (4) cytology with 75 lymphocytes and 10 monocytes (**Figure 1D**); (5) elevated CSF cytokines (IL-6: 25.6 pg/ml normal ≤ 7, IL-8: 155.3 pg/ml normal ≤ 62, IL-10: 51.5 pg/ml normal ≤ 9.1, TNF-α: 17 pg/ml normal ≤ 8) but normal blood cytokines; (6) CSF mNGS detected herpesvirus 7 (HHV-7, 150 sequences) and Epstein-Barr virus (EBV, 13 sequences) with high confidence (Testing Results from V-Medical Laboratory Co., Ltd.: Viruses were kept only if they were found >5-fold in samples than in controls; the threshold criteria for viruses were based on the detection of nonoverlapping reads from ≥3 distinct genomic regions); (7) equal CSF and serum OBs, with local IgA synthesis in CSF but no blood-brain barrier dysfunction; (8) negative neural autoantibodies in CSF and serum. Serum autoantibody testing at relapse demonstrated isolated re-elevation of thyroid autoantibodies (anti-Tg 292 IU/mL, anti-TPO 270 IU/mL) with normal thyroid function, whereas the anti-SSA and anti-SSB antibodies were negative. The brain MRI revealed a new T2-FLAIR hyperintensity in the right periventricular white matter compared with previous imaging (**Figures 2I, J**). The EEG was normal (**Figure 3C**).

The recurrence of similar clinical manifestations led us to reinstate the previously effective treatment. Treatment was initiated on admission with IVIG (0.4 g/kg/day for 5 days) and ganciclovir (5 mg/kg every 12 hours). On day 3, the patient developed acute mania characterized by disinhibited behavior and impaired impulse control, necessitating physical restraint and sedation with diazepam, phenobarbital, and dexmedetomidine. Due to ongoing deterioration, methylprednisolone was initiated at 500 mg/day × 3 days, then tapered by half every 3 days (240 → 120 mg/day) to oral 60 mg/day. Clinical improvement began on the fifth day. She was discharged on day 11 on a tapering regimen of oral methylprednisolone, reduced by 4 mg biweekly until cessation.

One month after discharge, the patient developed palpitations and hand tremors; laboratory tests confirmed hyperthyroidism, which was treated with methimazole. At one-year follow-up, she remained free of neuropsychiatric symptoms and neurological deficits, and brain MRI showed decreased white matter hyperintensities (**Figures 2K, L**).

## Discussion

Initially, fever, headache, vomiting, and neck resistance suggested central nervous system infection. Routine blood tests revealed leukocytosis with neutrophilic predominance; comprehensive clinical evaluations found no evidence of bacterial infection. Based on the established literature indicating that acute cortical dysfunction typically presents as background slowing without δ-wave intrusion, whereas combined cortical-subcortical involvement frequently exhibits both features (10), the initial EEG—showing background slowing with intermittent focal δ waves—was highly suggestive of meningoencephalitis rather than isolated meningitis. Brain MRI also showed punctate T2-FLAIR hyperintensities in white matter. Although mNGS of CSF was negative in the first hospitalization, CSF analysis was more consistent with viral encephalitis than bacterial infection (**Table 1**). Given that common viral pathogens of encephalitis include HSV, varicella zoster virus (VZV), EBV, cytomegalovirus (CMV), and human herpesviruses 6/7 (HHV6/7) (11), and acyclovir was unavailable at our institution, ganciclovir—a drug with broad coverage against these viruses—was used as first-line empirical therapy. The patient’s condition improved with ganciclovir and other symptomatic treatments.

**Table 1:** Timeline summarizing the clinical course.

Hospitalization and follow-up	Day	Medical history; symptoms	CSF findings	MRI changes	Antibody titers	Treatments
First hospitalization	Day 1	4 days of high-grade fever, headache, nausea, and vomiting	Pressure: 215 mm H_2_O; Routine (normal < 8/uL): WBC 93/L (multinucleated cells 20/L, mononuclear cells 73/L); Biochemical protein 0.684 g/L; Cytology amount of cells >100: lymphocytes (59%), neutrophils (25%). and monocytes (16%, including 2% activated) (**Figure 1a**).	punctate T2-FLAIR hyperintensities in left periventricular and hemisphere white matter (**Figures 2a, b**)		Ganciclovir, Cefotaxime; Mannitol; Glycerin fructose
	Day 3	high-grade fever resolving	mNGS: Negative			
	Day 7	normal temperature; headache improvement				Discontinue mannitol, glycerol, fructose, and cefotaxime; Continue ganciclovir
	Day 11	Recovery	Pressure 120 mm H_2_O. Routine WBC 30/μL (multinucleated cells 1/μL. mononuclear cells 29/μL); Biochemical normal; Cytology: lymphocytes 77. monocytes 5 (**Figure 1b**)			
	Day 14	Discharge				Oral ganciclovir
22 days interval						
Second hospitalization	Day I	9 days of decreased responsiveness, 2 days of hypersomnia and 21 hours of mania	Pressure 200 mm H_2_O; Routine WBC 17/μL (mononuclear cells 17/μL) Biochemical normal; Cytology: lymphocytes 58 and monocytes 6 (**Figure 1c**)	increased hyperintensities in periventricular and hemispheric white matter (**Figures 2c, d**)		Phenobarbital for sedation; IVIG: 0.4 g/kg/day for 5 days
	Day4	mania improved; psychomotor slowing and memory impairment				Discontinue phenobarbital
	Day 6		Oligoclonal bands only existed in CSF (**Figure 1e**); elevated IgG index		In serum elevation of anti- Tg (1407 IU/mL) and anti- TPO (657 IU/mL) antibodies; In serum: elevation of anti- SSA (56.- 4 IU/mL), anti-SSB (123 IU/mL), and antinuclear antibodies (123 IU/mL) Neural autoantibodies in CSF and serum: Negative	Methylprednisolone therapy: 500 mg-240 mg- 160 mg-80 mg, each administered for 3 days
	Day 15	psychomotor slowing and memory impairment; transient mania		punctate hyperintensities reduced (**Figures 2e, f**)		Added oral piracetam
	Day 18	transient mania improved				Piracetam: discontinue; Methylprednisolone: 40mg/day orally
	Day 21	Discharge				Methylprednisolone: 40 mg/day orally, 2 weekly 4 mg tapers
3-month follow-up	3-month	complete recovery		punctate hyperintensities in hemisphere white matter gradually dissipated (**Figures 2g, h**)	anti-SSA and anti-SSB antibodies: Negative anti-Tg and anti-TPO: Negative	Continue methylprednisolone orally with a taper
19 months interval						
Third hospitalization	Day 1	7 days of low-grade fever (peak 37.5°C) and headache, 4 hours of decreased responsiveness	Pressure 165 mm H.O. Routine WBC 36/μL (multinucleated cells 1/μL, mononuclear cells 35/μL); Biochemical normal; Cytology: lymphocytes 75, monocytes 10 (**Figure 1d**)	a new T2-FLAIR hyperintensity in the right periventricular white matter (**Figures 2i, j**)		Ganciclovir, IVIG: 0.4 g/kg/day for 5 days
	Day 3	acute mania	mNGS HHV-7 (150 sequences). EBV (13 sequences); equal CSF and serum OBs		In serum elevation of anti- Tg (292 IU/mL) and anti- TPO (270 IU/mL); In serum anti-SSA and anti- SSB antibodies negative; Neural autoantibodies in CSF and serum Negative	Sedation with diazepam, phenobarbital, and dexmedetomidine; Methylprednisolone: 500 mg-240 mg-120 mg, each administered for 3 days
	Day 5	Beginning clinical improvement				
	Day 11	Discharge				Methylprednisolone: 60 mg/day orally, 2 weekly 4 mg tapers
1-year follow-up	1 month	Developed hyperthyroidism				Methimazole
		Continue recovery without relapse		white matter hyperintensities decreased (**Figures 2k, l**)		

Abbreviations: CSF, cerebrospinal fluid; MRI, magnetic resonance imaging; mNGS, metagenomic next-generation sequencing; IVIG, intravenous immunoglobulin; anti-Tg, anti-thyroglobulin; anti-TPO, anti-thyroid peroxidase; SS, Sjögren's syndrome; HHV-7, human herpesvirus 7; EBV, Epstein-Barr virus.

22 days after recovery, the patient was readmitted with new-onset psychiatric symptoms, which marked the second distinct encephalitic episode, raising the clinical consideration of bimodal encephalitis. A diagnosis of probable autoimmune encephalitis was considered according to established diagnostic criteria (12) (**Table 2**), supported by the following patient findings: (1) acute psychiatric symptoms without prior psychiatric history; (2) CSF abnormalities including pleocytosis, oligoclonal bands, and elevated IgG index; (3) progressive T2-FLAIR white matter hyperintensities involving paraventricular and subcortical regions; and (4) exclusion of other plausible causes. Oligoclonal bands restricted to CSF and the elevated IgG index indicated the existence of intrathecal immunity, and the patient’s significant clinical improvement following immunotherapy with IVIG and methylprednisolone pulse provided further corroborative evidence for the autoimmune phase of the illness. However, standard neural antibody panels in both serum and CSF were negative, which ruled out common autoimmune encephalitis. Serological profiling uncovered evidence of broad systemic autoimmunity, with elevated antibodies against SSA, SSB, thyroglobulin, and thyroid peroxidase (**Table 1**). Can these antibodies be responsible for the autoimmune etiology?

**Table 2:** 2016 Graus et al. revised diagnostic criteria for probable autoimmune encephalitis.

Diagnostic criteria for possible autoimmune encephalitis (met all of the three following criteria)
1. Subacute onset (progression of <3 months) of:
①working memory deficits (short-term memory loss); ②altered mental status (decreased or altered level of consciousness, lethargy, or personality change); ③or psychiatric symptoms.
2. At least one of the following:
① New focal central nervous system (CNS) findings. ② Unexplained seizures (not due to a prior seizure disorder). ③ CSF pleocytosis (white blood cell > 5/mm³) or oligoclonal bands. ④ MRI: T2-FLAIR hyperintensities highly restricted to the medial temporal lobe(s) (limbic encephalitis) or multifocal lesions involving grey/white matter, suggestive of demyelination or inflammation.
3. Reasonable exclusion of alternative causes.

The antibodies against SSA and SSB suggested a possible link to Sjögren’s syndrome (SS), which has complex and diverse extraglandular manifestations. The prevalence of neurological manifestations in primary SS is estimated to be around 20% (13) affecting both the peripheral nervous system (PNS) (14) and the central nervous system (CNS). In 1983, Alexander and colleagues first reported five cases of acute meningoencephalitis (AME) in patients with SS (15). CNS involvement in SS encompasses a spectrum of manifestations, ranging from focal/multifocal or diffuse lesions and MS-like demyelinating disease to aseptic meningitis (16) (17–20). As positivity for anti-SSA/SSB antibodies alone is insufficient to meet the American-European Consensus Group (AECG) criteria for Sjögren’s syndrome (SS) (21) (22), the diagnosis could not be confirmed. Regrettably, the patient declined further evaluation with a salivary gland biopsy when recovering from her psychiatric symptoms. Although the absence of sicca symptoms (xerostomia and xerophthalmia) alone, without performing other specific investigations (e.g., Schirmer test and salivary gland ultrasound), does not completely exclude SS, the fact that the patient’s anti-SSA/SSB antibodies not only normalized during the recovery phase but also did not re-elevate during relapse led us to abandon SS as a diagnostic consideration.

In addition to the elevated anti-SSA/SSB antibodies, anti-Tg and anti-TPO antibodies linked to Hashimoto’s encephalopathy (HE) were also increased. The dynamic changes of serum anti-Tg and anti-TPO antibody titers—correlating with the patient’s relapsing-remitting course—make it difficult for us to ignore the existence of HE. According to the HE diagnostic criteria, the patient met the following requirements: 1) encephalopathy with altered mental status, confusion, hallucinations, and delusions; 2) progression to hyperthyroidism ultimately; 3) brain MRI with non-specific abnormalities; 4) presence of serum anti-Tg and anti-TPO antibodies; 5) absence of well-characterized neuronal antibodies in serum and CSF; and 6) reasonable exclusion of alternative causes (8, 12). The hallmark presenting feature of HE is a nonspecific encephalopathy with altered mental status and consciousness (confusion to coma) and cognitive impairment. CSF anti-thyroid antibody testing was not conducted due to early diagnostic limitations and restricted laboratory capacity; however, this test is not required by current diagnostic criteria. The presence, not the level, of serum thyroid antibodies is key. Thus, HE, also called steroid-responsive encephalopathy associated with autoimmune thyroiditis, should be considered in encephalopathic patients even with mild antibody elevations, as these antibodies reflect HE’s autoimmune nature rather than direct pathogenicity (23). But antibody titers can be a marker of treatment response (24). High levels of thyroid autoantibodies along with antinuclear antibodies indicate broader autoimmune activation, as over 30% of HE patients have overlapping autoimmune conditions (e.g., SLE and Sjögren’s syndrome), offering a plausible explanation for the transient positivity of anti-SSA and anti-SSB antibodies (8, 25). HE pathogenesis remains unclear due to a lack of neuropathological data. Among the proposed mechanisms, autoimmune-mediated vasculitis resulting in cerebral hypoperfusion and edema-induced dysfunction is a leading hypothesis, supported by transient steroid response and limited histologic findings (26, 27). Although MRI in HE is nonspecific, typical findings include T2/FLAIR white matter hyperintensities (focal or confluent, punctate or patchy) that often improve with steroids (23, 28). A characteristic migratory pattern—new lesions emerging as others resolve—may be seen, sometimes evolving into confluent leukoencephalopathy. These features may help differentiate HE from other autoimmune encephalitides (29). CSF studies could show negative or mild abnormalities such as lymphocytic pleocytosis or slightly elevated protein levels with intrathecal synthesis of IgG or not (9, 30). The condition typically presents with a relapsing-remitting encephalopathy and responds well to steroids and IVIG (8, 31). The clinical and laboratory findings in this case closely mirrored those described in the literature. The patient’s thyroid function was normal during the first episode, as neuropsychiatric disorders can occur in euthyroid states (32). However, she eventually developed hyperthyroidism during the relapse stage.

What initiates this aberrant immune response? The biphasic pattern of the disease provides a compelling clue. Although initial CSF mNGS was negative, HHV-7 and EBV were detected at relapse, initiating a similar cascade marked by recurrent symptoms, new MRI white matter lesions, nonspecific neuroinflammation (elevated CSF cytokines), and rebound of anti-Tg/TPO antibodies (**Table 1**). Previous studies have shown that HHV-7 in pediatric CSF can cause meningoencephalitis with neuropsychiatric symptoms and demyelinating lesions, suggesting immune-mediated pathology (33). A separate case reported active HHV-7 CNS infection leading to acute seronegative autoimmune encephalitis in a 10-year-old girl (34). HHV-7 infection correlates significantly with neuro-specific antibodies (ns-Ab), highlighting its role in CNS autoimmunity (35). Primary HHV-7 infection usually occurs before age five (36). After initial infection, the virus establishes lifelong latency in specific immune cells, creating a persistent reservoir (37, 38), from which reactivation may occur during immune suppression or cellular activation (37). EBV, also detected in this case, is typically acquired in early childhood (>90% adult seroprevalence) and then persists latently in B lymphocytes with periodic reactivation (39, 40). Previous research has found a significant positive correlation between EBV nuclear antigen IgG and anti-TPO antibody levels, implicating EBV in the early immune activation of Hashimoto’s thyroiditis (41). The reactivation of EBV also can target myelin-related proteins to trigger or maintain the autoimmune responses (42). The above literature evidence, together with the detection of EBV and HHV-7 in the CSF, supports the hypothesis that reactivation of these latent viruses is associated with recurrent HE, presenting as relapsing bimodal encephalitis. However, a limitation of this case is that comprehensive virological profiling was incomplete, specifically, serological testing for EBV and HHV−7 antibodies, as well as DNA testing of HHV-7 and EBV in blood, due to the limitations of hospital testing capabilities and the limited early understanding of the disease.

## Conclusion

We present a rare adult case of relapsing bimodal encephalitis. Cerebrospinal fluid (CSF) metagenomic next-generation sequencing (mNGS) during the relapse definitively identified both HHV-7 and EBV. We propose that reactivation of these latent herpesviruses is likely associated with the autoimmune phase of the illness, as evidenced by acute neuropsychiatric onset, CSF inflammatory markers (pleocytosis, oligoclonal bands, elevated IgG index, elevated CSF cytokines), progressive T2-FLAIR white matter hyperintensities, and a clear response to immunotherapy. Notably, while standard neural autoantibody panels were negative throughout the clinical course, anti-Tg and anti-TPO antibodies exhibited a disease-correlated pattern, with titers rising concurrently with each relapse. This case illustrates that HHV-7/EBV reactivation may be associated with a recurrent, virally precipitated autoimmune encephalopathy—clinically presenting as relapsing bimodal encephalitis—through thyroid antibody-associated Hashimoto’s encephalopathy rather than conventional neuronal antibodies.

## Data Availability

The datasets presented in this study can be found in online repositories. The names of the repository/repositories and accession number(s) can be found in the article/**Supplementary Material**.
