# Supplementary figures and images for: Behind the mask of relapsing bimodal encephalitis: herpesvirus 7 and Epstein-Barr virus associated with Hashimoto’s encephalopathy: a case report
Source: Front Immunol. 2026 May 29;17:1782631. doi: 10.3389/fimmu.2026.1782631 (PMC13259914; doi:10.3389/fimmu.2026.1782631)

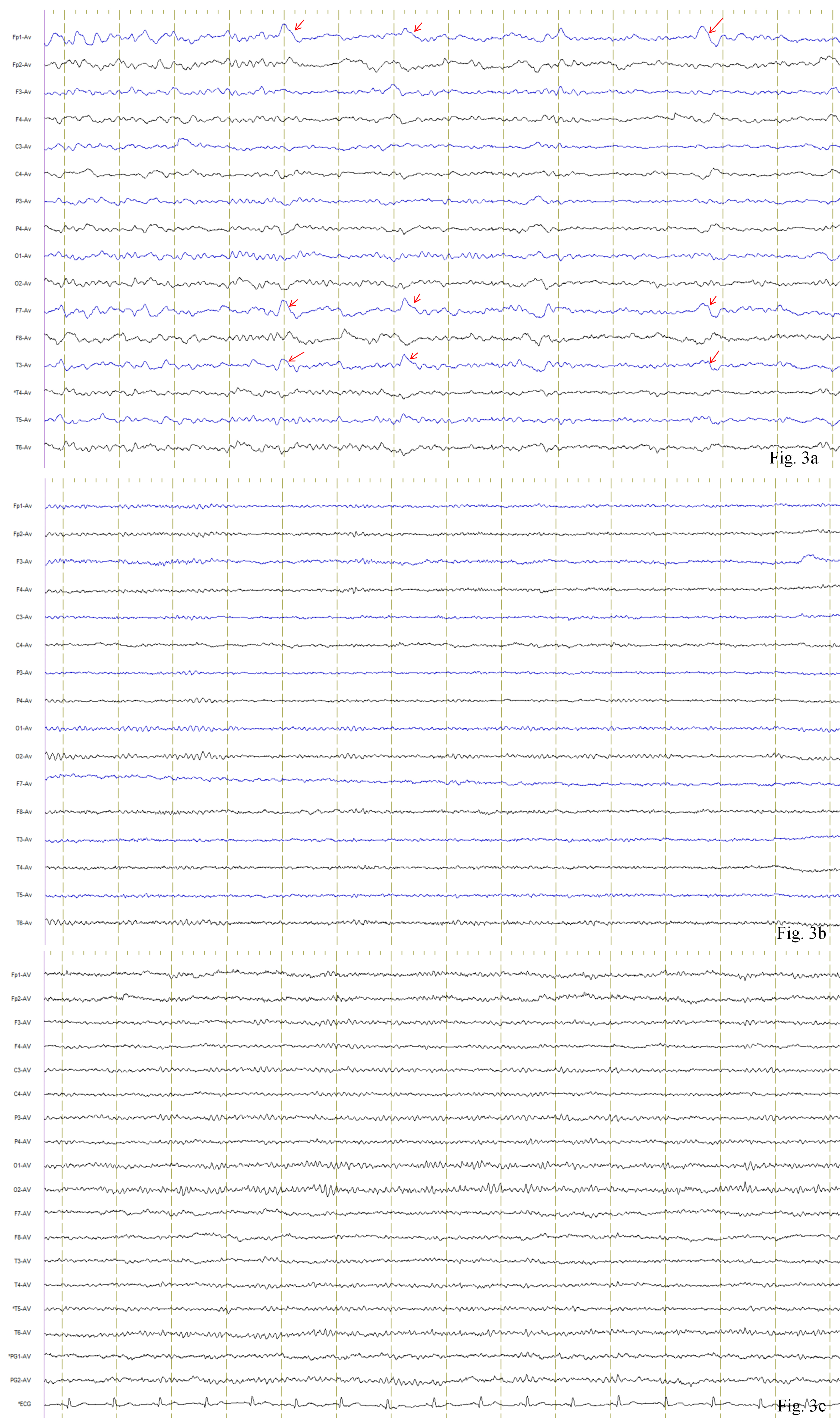

Supplement: Supplementary file 1 [file Image1.jpeg]

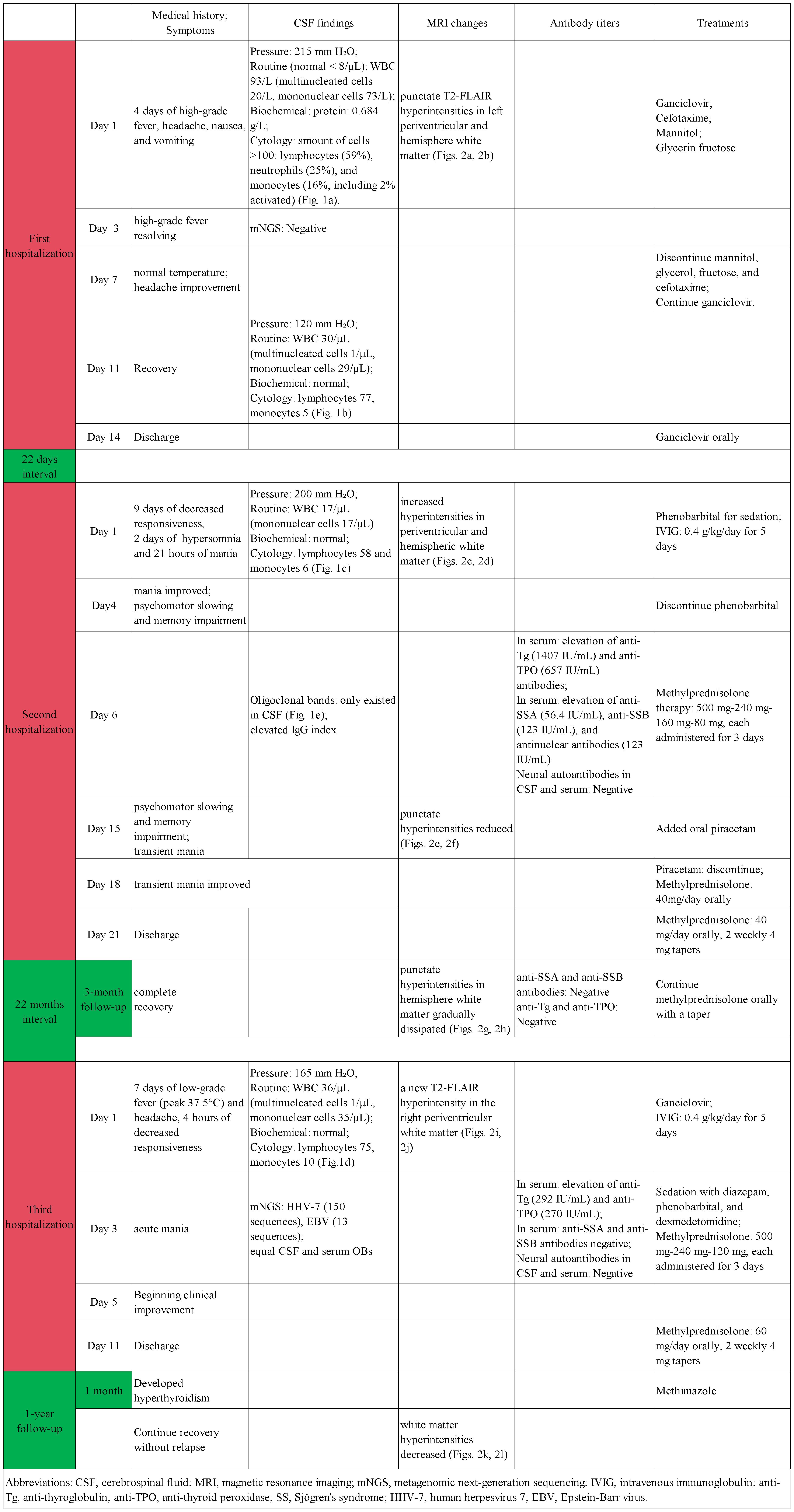

Supplement: Supplementary file 2 [file Image2.jpeg]
